# Granulocytic Myeloid-Derived Suppressor Cells as Negative Regulators of Anticancer Immunity

**DOI:** 10.3389/fimmu.2020.01963

**Published:** 2020-08-27

**Authors:** Elliot D. Kramer, Scott I. Abrams

**Affiliations:** Department of Immunology, Roswell Park Comprehensive Cancer Center, Buffalo, NY, United States

**Keywords:** polymorphonuclear myeloid-derived suppressor cells, antitumor immunity, immune suppression, immunotherapy, tumor progression

## Abstract

The immune system plays a critical role in cancer progression and response to therapy. However, the immune system can be compromised during the neoplastic process. Notably, the myeloid lineage, which gives rise to granulocytic cells, including neutrophils, is a well-recognized target of tumor-mediated immune suppression. Ordinarily, granulocytic cells are integral for host defense, but in neoplasia the normal process of granulocyte differentiation (i.e., granulopoiesis) can be impaired leading instead to the formation of granulocytic (or PMN)-myeloid-derived suppressor cells (MDSCs). Such cells comprise various stages of myeloid differentiation and are defined functionally by their highly pro-tumorigenic and immune suppressive activities. Thus, considerable interest has been devoted to impeding the negative contributions of PMN-MDSCs to the antitumor response. Understanding their biology has the potential to unveil novel therapeutic opportunities to hamper PMN-MDSC production in the bone marrow, their mobilization, or their effector functions within the tumor microenvironment and, therefore, bolster anticancer therapies that require a competent myeloid compartment. In this review, we will highlight mechanisms by which the neoplastic process skews granulopoiesis to produce PMN-MDSCs, summarize mechanisms by which they execute their pro-tumorigenic activities and, lastly, underscore strategies to obstruct their role as negative regulators of antitumor immunity.

## Introduction

Myeloid-derived suppressor cells (MDSCs) are cells of the immune system that are widely described as an immature subset arising from the myeloid lineage and defined by their ability to impede both innate and adaptive immunity, including against cancer (1–4). The suppressive activity of MDSCs has been demonstrated both *in vitro* and *in vivo* to inhibit the activation and expansion of adaptive T cell immunity, as well as to promote the growth and spread of cancer (5). Natural killer (NK) cells of the innate immune system are also susceptible to MDSC-mediated immune suppression through exposure to nitric oxide (NO) and TGF-β (6, 7). Broadly, in mouse models, MDSCs are divided into two main subsets: monocytic (M-MDSCs) and polymorphonuclear (PMN-MDSCs, also known as granulocytic MDSCs). M-MDSCs resemble monocytes and are phenotypically defined as CD11b^+^Ly6C^hi^Ly6G^−^. PMN-MDSCs resemble neutrophils and are defined as CD11b^+^Ly6C^lo^Ly6G^+^ (8). In humans, at least three distinct subsets have been identified, reflecting those of granulocytic (CD11b^+^CD33^+^CD15^+^CD14^−^HLA-DR^−^) or monocytic (CD11b^+^CD33^+^CD15^−^CD14^+^HLA-DR^lo/−^) origin, as well as a more immature state lacking expression of both granulocytic and monocytic markers (9).

In cancer, MDSCs are abundant both in the tumor microenvironment (TME) and the periphery, such as the spleen and blood. Interestingly, few if any MDSCs are found in lymph nodes. The abundance of MDSCs, and the PMN-MDSC subset in particular, in cancer patients has been demonstrated to directly correlate with a poorer prognosis, resistance to diverse therapies, and reduced overall survival in multiple solid tumor types (10–17). PMN-MDSCs drive tumor growth and progression by suppressing T cell activation, as well as enhancing tumor invasion and metastasis. PMN-MDSCs can directly inhibit the activity of CD8^+^ cytotoxic T lymphocytes (CTLs). Mechanisms of CD8^+^ CTL inhibition include: (1) expression of the immune checkpoint ligand, programmed death-ligand 1 (PD-L1); (2) mobilization of regulatory T cells; and (3) release of immunosuppressive mediators or signals such as interleukin-10 (IL-10), arginase-1 (Arg-1), and reactive oxygen species (ROS) (6, 18, 19). The release of such mediators within the TME fosters a chronic inflammatory response that promotes tumor growth akin to “an un-healing wound.”

PMN-MDSCs are also capable of inducing tumor progression independently of their impact on the immune system. Specifically, they facilitate tumor angiogenesis by the release of pro-angiogenic factors (20) and promote the epithelial-to-mesenchymal (EMT) transition, both critical steps in metastatic progression (21). Furthermore, PMN-MDSCs assist in degrading the extracellular matrix by releasing metalloproteases, such as MMP9, which supports the pre-metastatic niche (22). While both MDSC subsets are important in tumor progression, this article will focus on the PMN-MDSC subset reflecting the theme of this review series.

## Tumor-Driven Disruption of Granulopoiesis

Ordinarily, granulocytic populations develop in the bone marrow and have a high turnover rate once they are released into the circulation and, thus, must be replenished continuously through the process of granulopoiesis (23). As with all circulating immune cells, the ultimate precursor of granulocytic cells is the hematopoietic stem cell (HSC). In the process of granulopoiesis, the HSC mesenchymal progenitor population differentiates into common myeloid progenitors (CMPs) followed by differentiation into granulocyte-monocyte progenitor (GMPs). As its name suggests, the GMP further bifurcates into monocytic progenitors (MPs) or granulocytic progenitors (GP), the immediate antecedents to monocytes or granulocytes, respectively (24).

**Figure 1:**
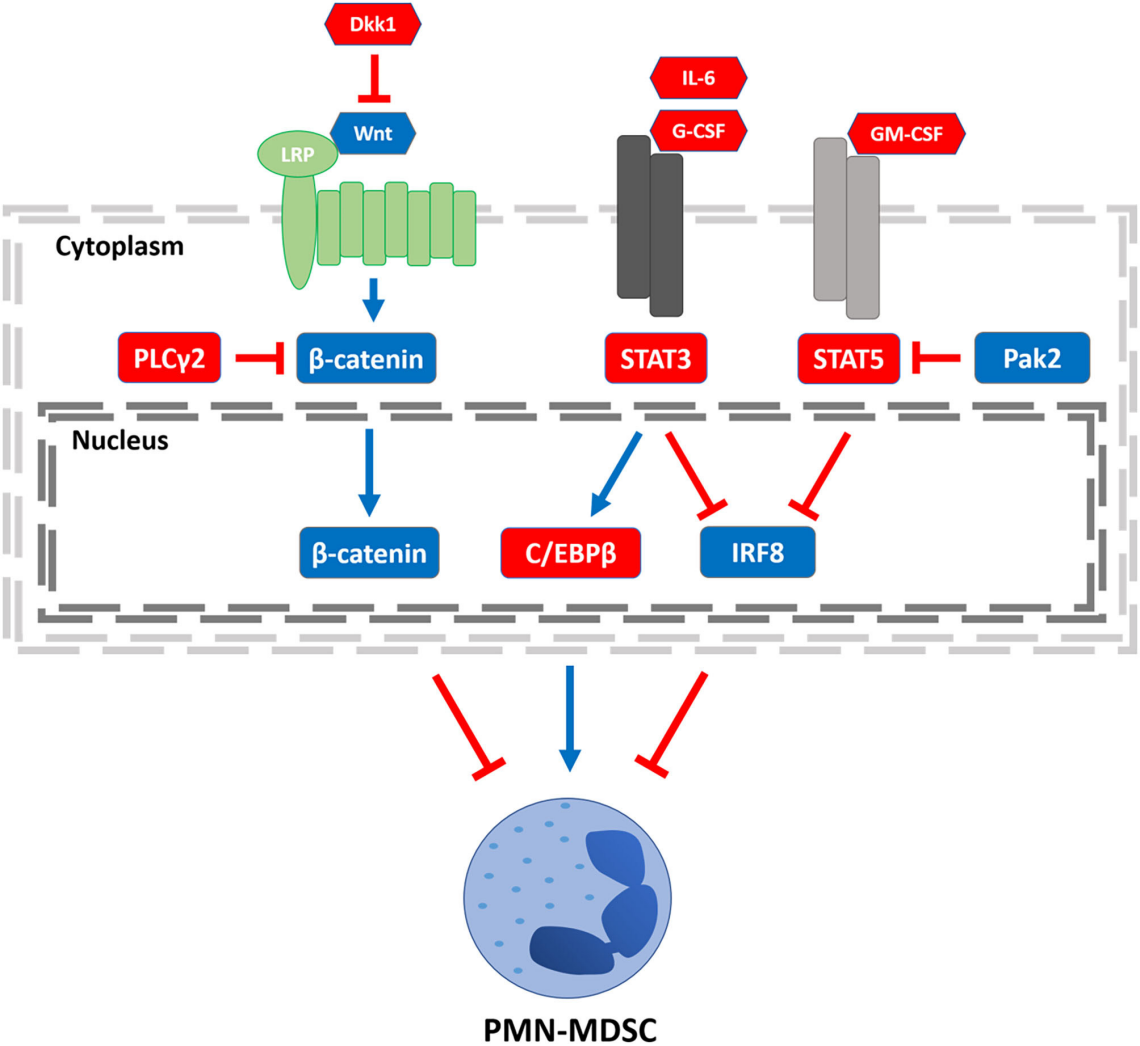
Transcriptional regulators of PMN-MDSCs. Three regulatory axes of PMN-MDSC development or function are depicted. Left to right: PLCγ2 and Dkk1 have been shown to decrease β-catenin signaling, increasing PMN-MDSC burden. STAT3 signaling can be activated by stromal- or tumor-derived factors such as IL-6 or G-CSF, enhancing C/EBPβ expression. STAT3 activation can also inhibit IRF8 expression. Both axes lead to an increase in PMN-MDSCs, although it remains to be determined whether there is crosstalk between the C/EBPβ and IRF8 pathways. Stromal- or tumor-derived GM-CSF engagement leads to STAT5 activation, which inhibits IRF8 expression, an effect that can be overcome by Pak2-mediated inhibition of STAT5. Legend: nodes shown in red or blue enhance or block PMN-MDSCs, respectively; arrows shown in red or blue inhibit or activate its downstream target, respectively.

However, in cancer, granulopoiesis is compromised, leading instead to the accumulation of PMN-MDSCs (25, 26). Each developmental junction in granulopoiesis is tightly choreographed by the differential expression of myelopoietic growth factors (e.g., G-CSF, M-CSF, or GM-CSF) and integral transcription factors. These “master regulators” are essential to the development and maturation of normal functional granulocytes, but in the face of tumor burden and the type and concentration of factors that tumors produce, these networks become dysregulated and impair myeloid differentiation driving the accumulation of PMN-MDSCs (27). Therefore, understanding the transcription factors that underlie MDSC development and function is crucial to improving the response of patients to immune-based therapies that require a competent myeloid compartment (28). In this review, we will highlight the important roles of several transcription factors known to drive PMN-MDSC production or function (Figure 1), including: interferon regulatory factor-8 (IRF8), signal transducer and activator of transcription (STAT) 3 or 5, CCAAT/enhance-binding protein-β (C/EBPβ), and β-catenin as well as their regulatory signals. While several transcription pathways are illustrated, the extent of crosstalk among them remains to be fully understood. We will then present future directions that exploit this knowledge toward the development of novel approaches to mitigate PMN-MDSC burden and improve antitumor responses.

## Transcriptional Regulators of PMN-MDSCs

### Interferon Regulatory Factor-8

Interferon regulatory factor-8 (IRF8) is a 48-kD protein containing two distinct N-terminal and C-terminal domains. The N-terminal domain contains the DNA binding site of the transcription factor, while the C-terminal domain contains the IRF association site (29). This IRF8 association domain (IAD) enables IRF8 to interact with other IRF family proteins, such as IRF1, IRF2, AP-1, or PU.1 (30). The heterodimerization of IRF8 with other binding partners determines the specific DNA binding motif recognized within the promoter regions of target genes and whether the binding of the particular IRF8 heterodimer to that region activates or represses target gene transcription (31).

IRF8 is expressed strongly by B cells and activated T cells of the lymphoid lineage and monocytes, macrophages, and dendritic cells (DCs) of the myeloid lineage (32–34). Indeed, IRF8 expression is essential for normal myelopoiesis in which it guides the differentiation of monocytic vs. granulocytic lineages at the GMP stage (35). At this stage, IRF8 acts as a positive regulator of monocytic and DC differentiation and as a negative regulator of granulocytic differentiation especially neutrophils (36, 37) This bifurcation point of the myeloid lineage is driven by the steady increase in the expression of IRF8 in MPs, and the reduction in the expression of IRF8 in GPs and eventually neutrophils. Indeed, in the global IRF8-deficient (IRF8^−/−^) model, mice exhibit profound alterations in hematopoiesis, with a marked skewing of myelopoiesis toward neutrophil production (38).

These neutrophils harbor an immature myeloid phenotype and, interestingly, have been shown to inhibit T cell activation; therefore, these IRF8-deficient myeloid cells seemed to mirror tumor-induced PMN-MDSCs at multiple levels (39). Furthermore, differential gene expression analyses revealed that IRF8-deficient myeloid cells displayed a gene expression profile that resembled tumor-induced PMN-MDSCs significantly more so compared to those of the non-tumor-bearing controls (39). Despite an increase in the total number of myeloid cells, the neutrophil bias comes at the expense of the Ly6C^+^ monocytic, CD8^+^ and CD103^+^ DC lineages. These shifts highlight the essential roles of IRF8 in moderating normal myelopoiesis and the generation of functional monocytic, DC, and granulocytic populations.

Previous work in our laboratory has demonstrated that IRF8 expression is reduced in both MDSC subsets and their progenitors in mouse models of mammary carcinoma (39, 40). These findings have been recapitulated by other investigators examining a number of solid tumors and hematologic malignancies (41–43). Tumor-driven downregulation of IRF8 was found to dysregulate myelopoiesis at least in part by promoting the expansion of tumor-derived GPs, which selectively led to PMN-MDSCs. Importantly, these tumor-derived GPs were shown to mirror the transcriptional profile of GPs isolated from IRF8^−/−^ mice compared to those of the non-tumor-bearing controls, lending further evidence that the loss of IRF8 expression during granulopoieses skews myeloid differentiation toward PMN-MDSCs. In a reciprocal manner, Netherby et al. (40) found that enforcing overexpression of IRF8 mitigated the expansion of GPs in these same mammary tumor models. On a functional level, Waight et al. (39) demonstrated that enhancing IRF8 expression in the myeloid compartment using a tissue-specific (CD11b) IRF8-transgenic mouse model led to a decrease in the abundance of PMN-MDSCs and a significant improvement in the antitumor response to anti-CTLA-4 immune checkpoint inhibitor therapy. Furthermore, a negative correlation between the expression of IRF8 and the abundance of the immature CD33^+^HLA-DR^−^ MDSC subset in breast cancer patients substantiated the important role of IRF8 as a negative regulator of PMN-MDSC levels originally observed in mouse tumor models.

### STAT3, STAT5, and C/EBPβ

Two negative regulators of IRF8 expression are the STAT3 and STAT5 proteins belonging to the highly conserved STAT family of transcription factors, which have been well-described as mediators of cellular immunity, differentiation, and cell cycle regulation (44). The STAT family is characterized by four unique domains: the Src homology 2 (SH2), C-terminal transactivation, coiled-coil, and DNA-binding regions that mediate their activation, dimerization, nuclear translocation, and DNA-binding, respectively. Canonically, STAT3 and STAT5 are activated by a cytokine receptor-associated Janus kinase (JAK), which leads to the phosphorylation of the STAT3 or STAT5 transactivation domain and subsequent dimerization and translocation to the nucleus (45, 46). Work in our laboratory identified STAT3 activation downstream of the G-CSF receptor (G-CSFR), as a previously unrecognized negative regulator of IRF8 expression and a driver of PMN-MDSC accumulation (39). STAT3 signaling has also been observed by other groups to promote PMN-MDSC function by enhancing Arg-1 expression (47). Inhibiting STAT3 signaling abrogated the function and development of the CD33^+^CD11b^+^CD15^+^ PMN-MDSCs (48).

Inhibition of STAT3 using the multitargeted tyrosine kinase inhibitor (TKI) Sunitinib has been shown to decrease MDSC burden in mouse renal cell carcinoma models (49). In addition to Sunitinib, Dasatinib, a broader spectrum TKI, has been shown to target and reduce M-MDSCs in CML patients. In other cancer models, however, while Dasatinib was able to modulate MDSCs, it strongly negatively impaired T cell activity which outweighed its potential use for immunotherapy (50–52). Promotion of angiogenesis via the release of vascular endothelial growth factor (VEGF) is one mechanism by which MDSCs exert their pro-tumorigenic effects in the TME. For example, in head and neck squamous cell carcinoma (HNSCC), inhibiting STAT3 was shown to decrease the production of VEGF by MDSCs, although the study did not delineate between monocytic and PMN subsets (47). Furthermore, active phosphorylated STAT3 is highly expressed in the human CD33^+^HLA-DR^−^ MDSC subset from the peripheral blood of metastatic melanoma patients when compared to healthy donors (26, 53). Addition of the STAT3 inhibitor AG490 alone was sufficient to significantly reduce MDSC-mediated T cell suppression and pro-angiogenic cytokine production in murine and human xenograft HNSCC models, respectively (54). In the study by Vasquez–Dundell (47) noted above, STAT3 inhibition using the novel compound Stattic decreased MDSC suppressive function by abrogating Arg-1 expression in MDSCs from patients with HNSCC. We established that at least one mechanism by which STAT3 facilitates PMN-MDSC expansion in cancer models is IRF8-dependent by demonstrating a unique G-CSF-STAT3-IRF8 axis (39). Thus, STAT3 is an important driver of the PMN-MDSC response by regulating both immunosuppressive and pro-angiogenic factors, as well as their development by inhibiting the expression IRF8.

In addition to its effects on IRF8 regulation, STAT3 activation has been shown to impact additional transcription factors important in PMN-MDSC biology; e.g., C/EBPs (55). C/EBPs are a family of transcription factors important in various cell functions, including metabolism, differentiation, and proliferation (56, 57). However, C/EBPβ in particular has been implicated as a key transcriptional regulator of emergency granulopoiesis (58). In a mouse sepsis model, PMN-MDSC numbers were significantly diminished in both early and late disease using a myeloid-specific *LysM*–cre conditional knockout of C/EBPβ (59). In the context of cancer, *Tek*-cre conditional knockout of C/EBPβ significantly decreased the abundance of CD11b^+^Gr-1^+^ PMN-MDSCs in the spleens of a mouse model of sarcoma (60). Furthermore, deletion of C/EBPβ in this study led to a decrease in the expression of the immunosuppressive factors Nos2, which encodes for inducible nitric oxide synthase (iNOS), and Arg-1. Interestingly, long non-coding C/EBPβ RNA (lnc-C/EBPβ), which inhibits C/EBPβ transcriptional activity by binding to the LIP isoform, has been shown to preferentially promote PMN-MDSC expansion (61, 62). A STAT3-C/EBPβ axis has been shown to impact PMN-MDSC biology in inflammation and cancer, but the role of non-coding RNAs and whether this axis interfaces with IRF8 requires further examination.

The STAT5 transcription factor is reported to be downstream of GM-CSF receptor engagement (63). GM-CSF is an important signaling molecule described to mediate the maturation and function of myeloid cells, including neutrophils (64). Recent work by Zeng et al. (65) uncovered a key role for the p21-activated kinase 2 (Pak2) in mediating the production of PMN-MDSCs. Their work elucidated a myeloid skewing of hematopoiesis in the *Mx1*-cre-driven Pak2 conditional knockout model. In this knockout model, a significant increase in granulocytes *in vivo* was observed. Further work (66) determined that these granulocytic cells were indeed PMN-MDSCs based on their T cell suppressive behavior, and that the overall number and suppressive function of PMN-MDSCs from the conditional Pak2 knockout host was greater than that of the wild-type controls. Importantly, they identified that Pak2-deficiency sensitized myeloid progenitors to GM-CSF stimulation, which led to the accumulation of PMN-MDSCs. This GM-CSF sensitivity was shown to increase the activity of STAT5 and decrease IRF8 expression, in agreement with the work of our laboratory (39). Therefore, enhancing Pak2 activity in myeloid progenitors may serve as a therapeutic target for future strategies aimed at increasing IRF8 expression and decreasing PMN-MDSC accumulation in cancer.

### β-catenin

β-catenin protein is a widely expressed and highly conserved protein characterized by its importance as a transcription factor that regulates cellular differentiation and proliferation. β-catenin is readily expressed in self-renewing tissues such as the epithelium of the gut, base of the hair follicle, the bone matrix, and hematopoietic bone marrow, but its activity is tightly controlled by the β-catenin degradation complex (67). This complex is an assembly of proteins that facilitate the phosphorylation and then ubiquitination and degradation of β-catenin. Canonical β-catenin signaling is activated after Wnt family ligands bind to the Frizzled receptor. This, in turn, leads to the phosphorylation of the β-catenin inhibitory complex and its subsequent degradation. Destruction of the inhibitory complex then stabilizes β-catenin in the cytosol. Free of negative regulation, β-catenin can then translocate to the nucleus and associate with transcriptional co-factors to drive the expression or repression of target genes (67). Constitutive activation of β-catenin signaling due to genetic mutations has been shown to induce neoplastic growth in cancers such as colorectal adenocarcinoma (68). β-catenin signaling also plays an important role in the normal differentiation of myeloid progenitors (69, 70).

Dysregulation of β-catenin signaling in myeloid cells has been implicated in promoting PMN-MDSC expansion in cancer (71). Capietto et al. (72) demonstrated that phospholipase Cγ2 (PLCγ2) drives β-catenin expression in PMN-MDSCs and that together, this PLCγ2-β-catenin axis inhibits the accumulation and suppressive phenotype of PMN-MDSCs in mouse models of lung carcinoma and melanoma. This same group went on to identify Dickkopf-related protein1 (Dkk1), a circulating soluble inhibitor of β-catenin activity, as a key source of β-catenin downregulation in their mouse tumor models (73). They extended these findings to cancer patients by correlating increased Dkk1 expression with an increase in PMN-MDSC abundance in pancreatic carcinoma samples. Furthermore, depletion of Dkk1 using an anti-Dkk1 antibody was sufficient to decrease tumor burden in their mouse tumor models; importantly, there was no additional significant impact on tumor growth following anti-Gr-1 antibody treatment suggesting that β-catenin mediated its effects through PMN-MDSCs.

The role of β-catenin in PMN-MDSC biology was further strengthened by Qian et al. (74) who showed that Cullin 4B (CUL4B), a scaffold protein known for its role in epigenetic repression of tumor suppressor genes, along with AKT and β-catenin negatively regulated PMN-MDSC numbers and function. Utilizing the *Tek*-cre conditional knockout system to selectively delete CUL4B in endothelial and hematopoietic cells, their work further demonstrated that the conditional deletion of CUL4B in these cells decreased β-catenin activity in PMN-MDSCs. This decrease in β-catenin led to an increase in PMN-MDSC accumulation and immune suppressive activity. As with the C/EBPβ pathway, it remains to be determined whether there is crosstalk between the β-catenin and IRF8 pathways. Additionally, β-catenin signaling is important in maintaining CD8^+^ T cell stemness and central memory responses, as well as enabling T cell tumor infiltration (75, 76). Thus, β-catenin signaling in the immune system likely plays important roles in multiple capacities – on the one hand, acting as a negative regulator of PMN-MDSCs and, on the other hand, acting as a positive regulator of T cell functionality. Understanding the β-catenin pathway in PMN-MDSC, as well as T cell biology may reveal novel therapeutic targets to enhance the efficacy of anti-cancer therapies.

## Anti-MDSC-Targeted Therapies in Human Cancers

While depleting or inactivating mouse PMN-MDSCs *in vivo* has been effective and has enhanced antitumor activity, particularly in combination regimens, therapies aimed at targeting human PMN-MDSCs *in vivo* have yet to similarly improve overall survival in patients (77). The prospect of combining PMN-MDSC-depleting or -inactivating strategies with other immune stimulatory agents, such as tumor vaccines or immune checkpoint inhibitors (ICIs), are a potentially effective way to concurrently mitigate PMN-MDSC involvement and heighten the efficacy of the antitumor immune response. The following is a brief summary of current anti-MDSC therapies that target and interfere with MDSC responses through different strategies.

### Cell Depletion

Currently, there are two active clinical trials (NCT01803152), and (NCT02544880), which involve gemcitabine and tadalafil to deplete MDSC populations, respectively (Table 1). Gemcitabine is a chemotherapeutic agent known to inhibit DNA synthesis and, thus, the expansion of dividing cells (91). Interestingly, gemcitabine has been observed to also diminish MDSC abundance in both mouse models and patients, although the precise mechanism of action is not entirely clear (78, 92, 93). Tadalafil is known to inhibit phosphodiesterase-5 which, interestingly, target PMN-MDSC effector mechanisms, iNOS and Arg-1 expression (79, 80). In addition to the gemcitabine studies, the novel small molecule RGX-104 that targets the liver-X receptor is being tested in combination with existing ICIs or chemotherapeutics (NCT02922764). This agent has been shown to deplete MDSCs by apoptosis in non-small cell lung cancer (81, 82). The tri-specific anti-CD16/IL-15/CD33 fusion protein GTB-3550 is being explored as a potential intervention for MDSC depletion by antibody-dependent cellular cytotoxicity (NCT03214666) (83).

**Table 1:** Clinical Trials Therapeutically Targeting MDSCs.

**Mechanism**	**Trial Title**	**Intervention(s)**	**Trial No./reference^**a**^**
Depletion	Dendritic cell vaccine with or without gemcitabine pre-treatment for adults and children with sarcoma	Dendritic cell vaccine, lysate of tumor, gemcitabine, imiquimod	NCT01803152 (78)
Depletion	PDE5 inhibition via tadalafil to enhance anti-tumor mucin 1 (MUC1) vaccine efficacy in patients with HNSCC	Tadalafil, anti-MUC1 vaccine	NCT02544880 (79, 80)
Depletion	A Study of RGX-104 in patients with advanced solid malignancies and lymphoma	RGX-104, nivolumab, ipilimumab, docetaxel, pembrolizumab, carboplatin, pemetrexed	NCT02922764 (81, 82)
Depletion	GTB-3550 (CD16/IL-15/CD33) tri-specific killer engager (TriKE™) for high risk heme malignancies	GTB-3550 TriKE	NCT03214666 (83)
Signaling	Ibrutinib and nivolumab in treating participants with metastatic solid tumors	Ibrutinib, nivolumab	NCT03525925 (84)
Signaling	Histamine receptor 2 antagonists as enhancers of anti-tumor immunity	Ranitidine	NCT03145012 (85)
Signaling	Myeloid-derived suppressor cells (MDSCs) in OSCC patients	β-glucan	NCT04387682 (86)
Signaling	VX15/2503 in combination with ipilimumab or nivolumab in patients with head and neck cancer	VX15/2503, nivolumab, ipilimumab	NCT03690986 (87, 88)
Maturation	Ipilimumab and all-trans retinoic acid combination treatment of advanced melanoma	VESANOID, ipilimumab	NCT02403778 (89)
Maturation	Pembrolizumab and all-trans retinoic acid combination treatment of advanced melanoma	Pembrolizumab with all-trans retinoic acid	NCT03200847 (89)
Transcription	A study of MTL-CEBPA in combination with a PD-1 inhibitor in patients with advanced solid tumors (TIMEPOINT)	MTL-CEBPA, pembrolizumab	NCT04105335 (90)

a *Reference refers to the original publication(s) that supported the rationale for the indicated clinical trial*.

### Modulating Signaling Pathways

Overcoming CD8^+^ CTL unresponsiveness or exhaustion by blocking immune checkpoint receptor-ligand interactions has led to dramatic clinical outcomes in subpopulations of patients with various cancer types. However, not all patients respond or display durable antitumor effects; therefore, targeting PMN-MDSCs may constitute a strategy to bolster the efficacy of ICIs. To test this notion, NCT03525925 investigates the impact of ibrutinib on PMN-MDSC function, which is thought to decrease Bruton's tyrosine kinase activation and subsequent indolamine 2,3-dioxygenase mRNA expression in PMN-MDSCs (84), a well-known mechanism of T cell suppression. Interestingly, NCT03145012 seeks to repurpose the widely prescribed histamine receptor 2 inhibitor, ranitidine, to decrease PMN-MDSC burden (85). As an innovative approach, the dietary supplement, β-glucan, has shown promise in depleting PMN-MDSCs in preclinical models and is being actively investigated in oral squamous cell carcinoma (OSCC) (NCT04387682) (86). Another recent research direction has been targeting the signaling molecule Semaphorin 4D, which has been associated with PMN-MDSC recruitment or function (87, 88). These findings have led to the design of a clinical trial involving an anti-Semaphorin 4D monoclonal antibody, VX15/2503, combined with ICIs (NCT03690986).

### Enforcing Maturation

It is also reasonable to develop therapies that target PMN-MDSC precursors to potentially abrogate their generation or expansion prior to their mobilization to the TME. Therefore, while much attention has been drawn toward testing therapies that target PMN-MDSCs themselves or their effector mechanisms, there remains a larger gap in progress toward identifying effective approaches that target the process of PMN-MDSC production in the first place, perhaps, by altering transcriptional regulation. Studies have made use of existing anti-leukemic agents, such as all-*trans*-retinoic acid (ATRA) (NCT03200847 and NCT02403778), which has led to a reduction in MDSC numbers or their function; however, their clinical efficacy requires further investigation (77, 89, 94, 95). Yet, there is still a specific lack of targeted therapies that leverage knowledge of the differentially expressed transcriptional elements discussed in this review, which are needed to impact or redirect myeloid differentiation.

### Regulating Transcriptional Events

While directly targeting and enhancing the activity of transcription factors is highly desirable, the ability to do so pharmacologically has yet to be clinically realized. At present, there is an active clinical trial (NCT04105335), which directly targets a transcriptional regulator of PMN-MDSCs. This trial seeks to utilize a small activating RNA oligonucleotide agent to upregulate C/EBPα to decrease MDSC burden (90). Perhaps, additional approaches that target upstream or downstream nodes of relevant signaling pathways may be feasible and considered as therapeutic targets. For example, therapies that inhibit STAT3 activation (47, 49) have the potential to both increase IRF8 expression and decrease C/EBPβ expression, thereby mitigating PMN-MDSC burden. There is also published rationale for an interaction between β-catenin signaling and IRF8 expression (96), which may be achieved through the use of anti-Dkk1 antibody. Additional investigations into combination therapy approaches that decrease both PMN-MDSC burden and impede immune checkpoints are also warranted. In summary, understanding the factors and molecular mechanisms that drive dysregulated myelopoiesis, and granulopoiesis specifically given the predominance of PMN-MDSCs in cancer, will likely lead to the discovery of new therapeutic targets to mitigate the pro-tumorigenic effects of PMN-MDSCs.

## Conclusions

Polymorphonuclear leukocytes, namely neutrophils, are essential for mediating acute inflammation and defending against pathogenic insults. These terminally differentiated cells have a high rate of turnover and are, therefore, continuously replenished by granulopoiesis in the bone marrow. The chronic inflammatory nature of tumorigenesis can promote the aphysiologic release of cytokines, chemokines, and growth factors that compromise the otherwise tightly coordinated process of granulopoiesis likely through disruption of transcriptional regulation. This dysregulation of granulopoiesis then culminates in the production of PMN-MDSCs, which migrate via the bloodstream into the TME where they carry out their immune suppressive and pro-tumorigenic activities. Indeed, the abundance of PMN-MDSCs in neoplasia has been significantly correlated with poor prognosis and decreased efficacy of anti-oncologic therapies across diverse cancer types. Therefore, there is sound and compelling rationale for therapeutically targeting PMN-MDSCs in neoplastic disease. While several approaches have been developed and applied in clinical settings, there remains important opportunities to develop better and more effective PMN-MDSC-targeting therapies. An alternative or additional approach to abrogate the involvement of PMN-MDSCs may be attacking their production at their point-of-origin in the bone marrow. To achieve this goal, it is important to understand and build upon our current understanding of the transcriptional mechanisms that underlie PMN-MDSC generation from their progenitors. Exploiting this knowledge has the promise to unveil novel therapeutic options to improve cancer patient outcomes impacted by high PMN-MDSC burden.

## Author Contributions

EK and SA wrote and edited the manuscript.

## Conflict of Interest

The authors declare that the research was conducted in the absence of any commercial or financial relationships that could be construed as a potential conflict of interest.
